# *Mus musculus* deficient for secretory antibodies show delayed growth with an altered urinary metabolome

**DOI:** 10.1186/s10020-019-0077-2

**Published:** 2019-04-03

**Authors:** Kim R. Simpfendorfer, Nancy Wang, Dedreia L. Tull, David P. De Souza, Amsha Nahid, Andre Mu, Dianna M. Hocking, John S. Pedersen, Odilia L. C. Wijburg, Malcolm J. McConville, Richard A. Strugnell

**Affiliations:** 1 The Department of Microbiology and Immunology, The University of Melbourne, at the Peter Doherty Institute for Infection and Immunity, Melbourne, Australia; 2 Metabolomics Australia, Bio21 Institute, The University of Melbourne, Parkville, Australia; 3 Doherty Applied Microbial Genomics, Department of Microbiology and Immunology, The University of Melbourne, at the Peter Doherty Institute for Infection and Immunity, Melbourne, Victoria Australia; 4 Microbiological Diagnostic Unit Public Health Laboratory, Department of Microbiology and Immunology, The University of Melbourne, at the Peter Doherty Institute for Infection and Immunity, Melbourne, Australia; 5 Alfred Hospital, Monash University, Prahran, Australia; 6 Department of Biochemistry and Molecular Biology, Bio21 Institute, The University of Melbourne, Parkville, Australia; 7 Present address: The Feinstein Institute for Medical Research, 350 Community Drive, Manhasset, NY 11030 USA

**Keywords:** SIgA, Inflammation, Gut, Permeability, Urinary biomarker, Tissue resident memory T cells

## Abstract

**Background:**

The polymeric immunoglobulin receptor (pIgR) maintains the integrity of epithelial barriers by transporting polymeric antibodies and antigens through the epithelial mucosa into the lumen. In this study, we examined the role of pIgR in maintaining gut barrier integrity, which is important for the normal development in mice.

**Methods:**

Cohorts of *pIgR*^*−/*−^ mice and their wildtype controls were housed under Specific Pathogen Free (SPF) conditions and monitored for weight gain as an indicator of development over time. The general physiology of the gastrointestinal tract was analysed using immunohistochemistry in young (8–12 weeks of age) and aged mice (up to 18 months of age), and the observed immunopathology in *pIgR*^*−/*−^ mice was further characterised using flow cytometry. Urinary metabolites were analysed using gas chromatography-mass spectrometry (GC-MS), which revealed changes in metabolites that correlated with age-related increase in gut permeability in *pIgR*^*−/*−^ mice**.**

**Results:**

We observed that *pIgR*^*−/*−^ mice exhibited delayed growth, and this phenomenon is associated with low-grade gut inflammation that increased with ageing. The gross intraepithelial lymphocytic (IEL) infiltration characteristic of *pIgR*^*−/−*^ mice was redefined as CD8α^+^αβ^+^ T cells, the majority of which expressed high levels of CD103 and CD69 consistent with tissue resident memory T cells (T_RM_). Comparison of the urinary metabolome between *pIgR*^*−/−*^ and wild-type mice revealed key changes in urinary biomarkers fucose, glycine and Vitamin B5, suggestive of altered mucosal permeability. A significant increase in gut permeability was confirmed by analysing the site-specific uptake of sugar probes in different parts of the intestine.

**Conclusion:**

Our data show that loss of the secretory antibody system in mice results in enhanced accumulation of inflammatory IELs in the gut, which likely reflects ongoing inflammation in reaction to gut microbiota or food antigens, leading to delayed growth in *pIgR*^*−/−*^ mice. We demonstrate that this leads to the presence of a unique urinary metabolome profile, which may provide a biomarker for altered gut permeability.

**Electronic supplementary material:**

The online version of this article (10.1186/s10020-019-0077-2) contains supplementary material, which is available to authorized users.

## Background

The mucosal surface lining of the gastrointestinal tract (GIT) is composed of microvilli and villi that increase the surface area and allow for efficient nutrient absorption into the body. The folded epithelium represents the largest surface area of the body exposed to the external environment, and the challenge of maintaining function and protection of this barrier is significant.

The mucosal immune system has evolved to protect the epithelia against pathogens and reduce the pathologies that might be expected if the highly inflammatory bacterial pathogen-associated molecular patterns (PAMPs) make contact with their cognate cellular ligands, including Toll-like and nucleotide-binding oligomerisation domain (NOD)-like receptors. In humans, approximately 3 g of secretory IgA (SIgA) and SIgM is secreted into the mucosal lumen of the GIT each day (Conley and Delacroix 1987). In particular, SIgA has been considered as a major defence against pathogens seeking to enter the epithelium (Strugnell and Wijburg 2010). In addition, the secretory immune system may also mediate the ‘excretion’ of antigens from the sub-epithelial tissues (Kaetzel et al. 1991). This excretion occurs through both specific and ‘polyspecific’ SIgA which bind antigens on the basolateral side of the epithelia surface after transepithelial or paracellular movement of antigens and microbes through the epithelial barrier. In this excretion hypothesis, it is argued that the secretory antibody (SAb) performs a key function in depleting antigen from the systemic compartment. According to Lamm’s model (Kaetzel et al. 1991), SIgA-antigen complexes bind to the polymeric immunoglobulin receptor (pIgR) at the basolateral surface of the epithelium, triggering receptor-mediated endocytosis and subsequent exocytosis of antibody-antigen complexes from the apical surface into the GIT lumen.

In the absence of SAbs across the GIT, movement of gut-derived antigens into systemic sites becomes less controlled. We have previously shown that antigen-specific T cells undergo more proliferation in response to orally fed antigen in *pIgR*^−/−^ mice than wild-type B6 mice (Sait et al. 2007), suggesting that immunological tolerance may be impaired and/or proteins may become more readily accessible to the systemic immune system in the absence of SAbs. The detection of serum albumin in the saliva and faeces suggest protein leakage across the epithelial barrier (Johansen et al. 1999), a sign of increased permeability at such sites. Increased trafficking from the gut also extends to microbes in *pIgR*^−/−^ mice. We observed that uptake of dominant commensal bacterial species into the mesenteric lymph nodes (mLNs) is increased in pIgR-deficient mice (Sait et al. 2007), highlighting an important role for SAbs in maintaining the integrity of the gut barrier. The capacity for controlling microbial influx has significant implications for mucosal immunity against pathogens in the GIT. We have shown that SAbs in naïve mice provided substantial protection against *Salmonella* invasion of the gut (Wijburg et al. 2006). Orally administered *Salmonella* disseminated to the Peyer’s Patches and mLNs earlier and in greater numbers in *pIgR*^*−/−*^ mice, which also showed increased shedding of the bacteria in the faeces. Therefore, SAbs are functionally important for conferring resistance to gut infection as well as for reducing the transmission of pathologic gut bacteria.

To date, much of the attention has focussed on the role of the pIgR in infectious disease immunity and transmission (Uren et al. 2005), and there is considerably less research on the role of Sab in mucosal homeostasis. It is suggested that disruptions to mucosal homeostasis in mice lacking SAb could arise when environmental antigens that would normally be neutralised and removed from the lumen, lamina propria (LP) and epithelium by SIgA and pIgR, are retained or accumulated (Strugnell and Wijburg 2010). The downstream effects of the failure to neutralise and remove antigen could potentially include heightened activation of immune cells in the GIT following increased ‘pathogen’ sensing through PAMPs; this leads to immune activation, structural damage to the epithelial barrier due to inflammation, and subsequent loss of integrity of the epithelial barrier. Accordingly, several groups have shown *pIgR*^−/−^ mice are more likely to develop systemic Ab responses against normal flora and have increased numbers of antibody-secreting cells (ASCs) in gastric-associated tissues (Sait et al. 2007; Johansen et al. 1999; Uren et al. 2003), a phenomenon that may have broader implications on the health and development of the host.

This study aimed to further explore the role of the secretory immune system in limiting the inflammation and permeability of the GIT under homeostatic conditions. Our data show a relative delay in growth in *pIgR*^−/−^ mice compared with their wild-type counterparts, a phenomenon associated with increasing infiltration of intraepithelial lymphocytes (IELs) that were high in surface expression of CD103 and CD69 characteristic of tissue resident memory T cells (reviewed in (Carbone 2015)). Using advanced gas-chromatography mass spectrometry (GC-MS) analysis, we found that *pIgR*^−/−^ mice displayed significantly altered metabolite markers in the urine, suggesting increased translocation of gut luminal contents into the murine host. Further investigations through direct measure of gut permeability in mice lacking SAbs indicate that the urinary metabolome profile can be used as a non-invasive method for measuring gut permeability.

## Methods

### Mice

The colony of B6.*pIgR*^−/−^ mice used in this study were generated by targeted mutagenesis of C57BL/6 (B6) mice as described (Uren et al. 2003) and maintained in the animal facility of the Department of Microbiology and Immunology, The University of Melbourne. Control B6 mice were maintained in the same breeding facility. All mice were bred and housed under SPF conditions and received food and water *ad libitum*. All mice were age and sex-matched for each experiment. Mice were weighed at regular intervals from weaning. All animal experiments were approved by The University of Melbourne Animal Ethics Committee, and were compliant with the Prevention of Cruelty to Animals Act (1986) and the National Health and Medical Research Council (NHMRC) Australian Code of Practice for the Care and Use of Animals for Scientific Purposes (1997).

### Cell preparation and flow cytometry

The small intestine was excised, washed in PBS, cut open longitudinally and then into 2 cm pieces. Intestinal pieces were incubated at 37 °C for 1 h with gentle shaking in dissociation buffer (3% foetal calf serum and 5 mM EDTA in HBSS) to release IELs. The mixture was filtered through a 70 μm cell strainer and washed twice in FACS/EDTA buffer (0.1% bovine serum albumin and 5 mM EDTA in PBS). Cells were centrifuged in 37% isotonic Percoll (GE Healthcare) for 20 min at 900×g. Lymphocytes were harvested from the pellet at the end of the centrifugation, washed twice and resuspended in FACS/EDTA buffer. For flow cytometry, 100 μl of cell suspension was incubated with fluorochrome-conjugated antibodies for 30 min on ice. The following antibodies (clone) were purchased from BD Bioscience or eBioscience: CD45 (30-F11), CD4 (GK1.5), CD8α (53–6.7), TCRβ (H57–597), TCRγδ (GL3), CD103 (2E7), CD69 (H1.2F3). MAIT cell-specific MR1 tetramer was obtained from James McCluskey (Reantragoon et al. 2013; Corbett et al. 2014), The University of Melbourne. NKT cell-specific CD1d tetramer was obtained from Dale Godfrey (Hammond et al. 2001), the University of Melbourne. After staining, cells were washed twice in FACS/EDTA buffer and propidium iodide (Sigma) was added for exclusion of dead cells immediately before analysis using the LSRII flow cytometer (BD Bioscience). Calibration beads (BD Bioscience) were used for cell counting on the flow cytometer. FACS data was analysed using FlowJo software (TreeStar).

### Cytokine analysis

For collection of serum, peripheral blood was drawn by cardiac bleeding immediately after euthanasia. Blood samples were allowed to clot for at least 3 h at room temperature, and serum was separated following two rounds of centrifugation. Whole section of small intestine was homogenised in 2 ml RPMI (Life Technologies) and complete EDTA-free protease inhibitor (Roche) was added to inhibit protein degradation. Gut content and debris was removed by three rounds of centrifugation for collection of cytokine-containing supernatant. The concentration of IL-2, IL-4, IL-6, IL-10, IL-17A, IFN-γ and TNF was determined using the Mouse Th1/Th2/Th17 cytometric bead array (BD Bioscience) according to manufacturer’s instructions. Data was analysed using the FCAP Array software (BD Bioscience).

### GC-MS for metabolite analysis of urine

Urine (20 μL) was incubated with an equal volume of urease (20 μL, 0.5 units/μL in water, Sigma) at room temperature for 15 min to remove excess urea. Samples were then mixed with chilled ethanol (160 μL, containing 62.5 μM *scyllo*-inositol internal standard) to precipitate the urease and urinary proteins, for the extraction of urine metabolites. Samples were centrifuged (0 °C, 5 min. 14,000 rpm) and an aliquot of the resultant supernatant (20 μL) dried in vacuo prior to chemical derivatisation for GC-MS analysis. Chemical derivatisation and instrument conditions were as described in Long et al. (Long et al. 2015). Standard curves were produced for each sugar and quantification in mouse urine was achieved with the use of *scyllo*-inositol as an internal standard. The detection limit for the four sugars was calculated to be within the range of 100–300 pM/L. Inter- and intra-assay coefficients of variation were less than 4.5 and 10.2%, respectively. AnalyzerPro software (Spectralworks, Runcorn, UK) was used to process the raw data.

For discovery of biomarkers, 272 urine metabolites were aligned and normalised for multivariate statistical analysis. The spectra of all resolved peaks were annotated by comparison to commercially available electron impact mass spectral libraries such as the NIST (Http://www.nist.gov) or AMDIS (Http://chemdata.nist.gov/mass-spc/amdis). The standard compounds that are assumed present at detectable levels within urine, were measured.

### Measurement of gastrointestinal permeability using sugar probes

GI permeability was assessed using a method adapted from Meddings and Gibbons (Meddings and Gibbons 1998). To validate the system, gastric damage was induced by a single oral gavage of aspirin (250 mg/kg in 75% ethanol) via a 4 cm gastric needle under light anaesthesia using Penthrane (Abbot Laboratories). Two hours later, mice received a single oral 200 μL inoculation containing sucrose 120 mg/mL, mannitol 30 mg/mL, lactulose 60 mg/mL and sucralose 30 mg/mL (Sigma). Urine was collected at 1 to 4-h intervals for 24 h after inoculation of the sugars in 10% thymol in isopropanol. Samples, including *scyllo*-inositol which was used as an internal standard, were weighed, urease-treated and ethanol extracted before GC-MS analysis. All samples were analysed in duplicate using an Agilent 7890 gas chromatograph, coupled to a 5975C mass selective detector. GC separations were performed using a Varian VF-5 ms capillary column. Standard curves were produced for each sugar and quantification in mouse urine was achieved with the use of *scyllo*-inositol as an internal standard. The internal standard and sugar probes eluted as follows: *scyllo*-inositol 20.8 min, mannitol 19.9 min, sucrose 26.0 min, sucralose 26.6 min and lactulose 26.4 min. The detection limit for the four sugars was calculated to be within the range of 100–300 pM/L. Inter- and intra-assay coefficients of variation were less than 4.5 and 10.2%, respectively. AnalyzerPro software was used to process the raw data.

### Statistical analysis

Annotated urine metabolites were normalised by mean absolute deviation (MAD). To determine the relationship between samples, sparse partial least square discriminant analysis (sPLS-DA) was computed using the mixOmics R package (available through: mixomics.org/access/download). GraphPad Prism (v7) was used for statistical analyses; unpaired *t* test was used for comparison between two groups, or one-way ANOVA was used between three groups. The non-parametric Mann Whitney *U*-test was used for statistical analysis of ratios.

## Results

### The loss of SIgA is associated with pathology in the gut

The pIgR is responsible for secreting SAbs into the gut lumen. Comparable to earlier reports from our lab as well as others (Johansen et al. 1999; Uren et al. 2003; Shimada et al. 1999), our colony of *pIgR*^−/−^ mice exhibited a severe deficiency of SIgA in the faecal extract (Additional file 1: Figure S1A) and subsequently a significant accumulation of IgA in the serum (Additional file 1: Figure S1B), while no apparent deficiency was observed with IgG in either the faeces (Additional file 1: Figure S1C) or the serum (Additional file 1: Figure S1D). These data confirm that *pIgR*^−/−^ mice carry a deficiency of SIgA in the gut.

**Fig. 1:**
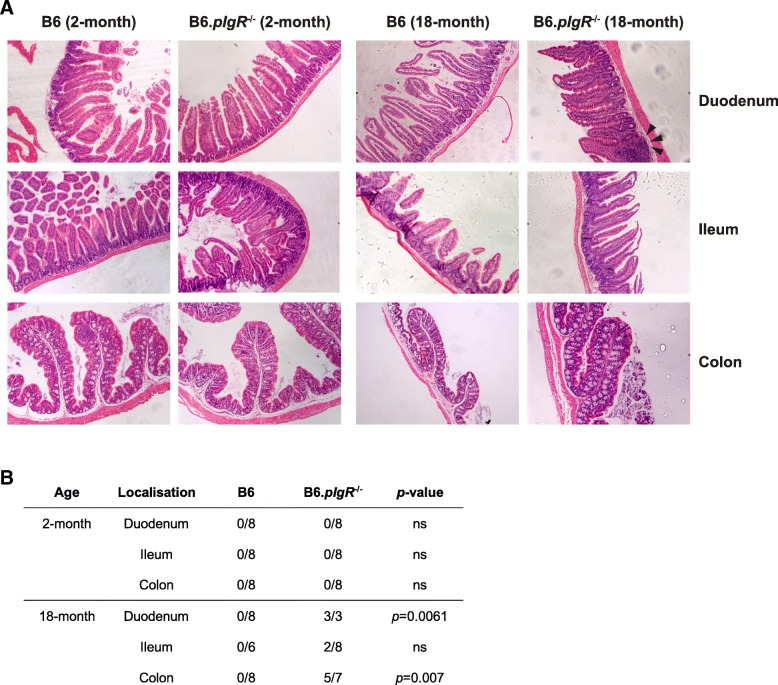
Age-dependent immunopathology in the GIT of *pIgR*^−/−^ mice. **a** Intestine sections of 2-month and 18-month old male B6 and *pIgR*^*−/−*^ mice were stained with H&E. Representative sections of duodenum, ileum and colon were imaged at 20× magnification. Arrows indicate pathology in duodenum of 18-month old *pIgR*^*−/−*^ mice. **b** Intestine sections were graded blindly by a pathologist. Intestines were described as normal or affected. Affected tissue exhibited a reduction in mucin, larger nuclei in crypt epithelium and seemingly increased number of lymphocytes. Affected samples are noted in the table. Statistical analysis was performed using a two-tailed Fisher’s exact test, ns indicates *p*-values > 0.05

It has been suggested that the deficiency of SAbs could affect the local intestinal environment through its role in regulating the microbiome (Reikvam et al. 2012; Rogier et al. 2014). However, we were unable to detect variations in the microbial community composition that could be attributed to the pIgR genotype using culture-independent techniques to profile the biodiversity of the terminal ileum (Sait et al. 2003). Alternatively, it is possible that SAbs maintain intestinal homeostasis by exerting a suppressive effect on inflammatory responses to commensal bacteria and common metabolites in the gut lumen. We found that while the GIT appeared healthy in young adult *pIgR*^−/−^ mice (2 months of age, Fig. 1a left panels), a range of abnormalities was observed in the duodenum and colon of 18-month-old *pIgR*^−/−^ mice (Fig. 1a right panels), with key pathological indicators including reduced mucin, larger nuclei in crypt epithelium and substantial lymphocytic infiltration in older animals. Such immunopathology was seen in a statistically significant proportion of *pIgR*^−/−^ mice at 18–24 months of age but was absent from age-matched B6 mice (Fig. 1b). These observations suggest that cumulative GIT damage takes place in the absence of SAbs and the pathology intensifies as mice age.

### Aged *pIgR*^−/−^ mice display an increase in T cell-mediated inflammation in the GIT

**Fig. 2:**
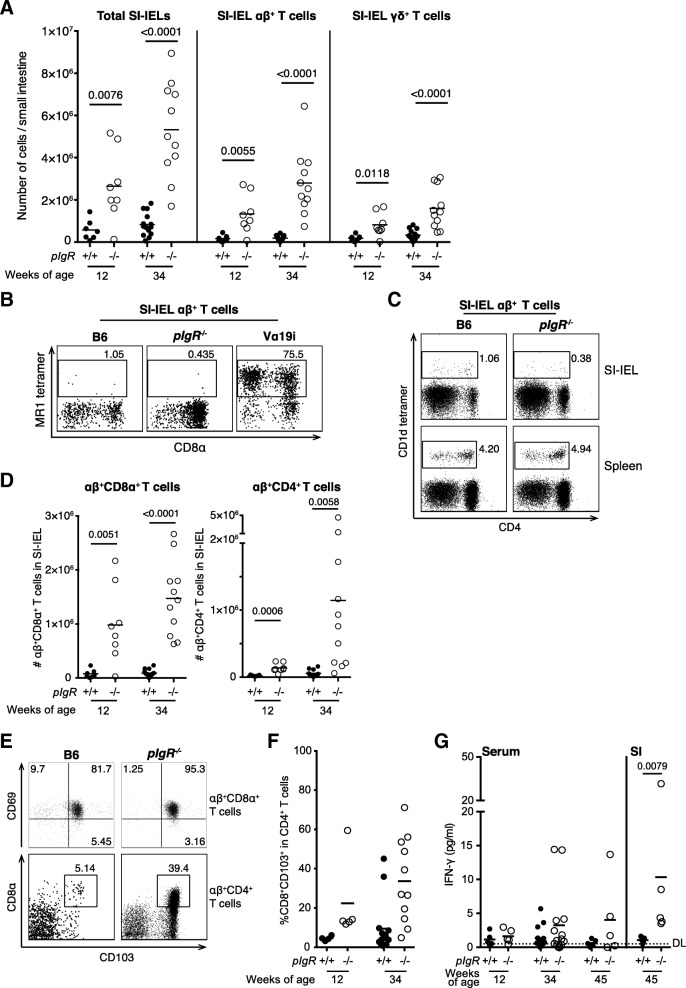
Flow cytometric analysis of small intestine intra-epithelial lymphocytes (IELs) reveals enrichment of αβ^+^ T cell subsets in older *pIgR*^*−/−*^ mice. IELs were collected from age- and sex-matched B6 (closed circle, 12-week *n* = 7, 34-week *n* = 14) and B6.*pIgR*^*−/−*^ (open circle, 12-week *n* = 8, 34-week *n* = 11) mice. **a** The total number of small intestine IELs (SI-IELs, CD45^+^), αβ^+^ T cells (TCRβ^+^) and γδ^+^ T cells (TCRγδ^+^) was increased in *pIgR*^*−/−*^ mice compared to B6 mice. Representative FACS profiles of SI-IEL αβ^+^ T cell are shown for **b** MAIT cells by MR1 tetramer staining with SI-IELs from Vα19i MAIT cell transgenic mice as positive control for gating (Kawachi et al. 2006), and **c** NKT cells by CD1d tetramer staining with splenocytes as positive control for gating. The number of **d** SI-IEL CD8α^+^αβ^+^ T cells (TCRβ^+^CD8α^+^CD4^−^) and SI-IEL CD4^+^αβ^+^ T cells (TCRβ^+^CD4^+^) was significantly increased in *pIgR*^*−/−*^ mice especially with age, and **e** representative FACS profiles for these subsets are shown. **f** Serum was collected from B6 and *pIgR*^*−/−*^ mice at indicated age and small intestine was homogenised in 2 ml RPMI. The concentration of IFN-γ was determined using mouse Th1/Th2/Th17 cytometric bead array (CBA). Dotted line indicates detection limit (DL). Data points indicate individual mice that were pooled from 3 to 4 independent experiments, mean ± SEM is shown. Unpaired *t*-test was used for statistical analyses and *p*-values are shown where < 0.05 is considered significant

The ‘excretory immune system’ hypothesis posits that the loss of SAbs results in an accumulation of antigens on the basolateral surface of the mucosal epithelia (Strugnell and Wijburg 2010; Robinson et al. 2001), leading to lymphoproliferation in the epithelium and lamina propria. Recently, several subsets of innate-like, invariant T cells, including natural killer T (NKT) cells (Middendorp and Nieuwenhuis 2009) and mucosal-associated invariant T (MAIT) cells, have been speculated to play a physiological role in the GIT. Whether a compensatory relationship exists between these T cells and SAbs remain to be determined. To characterise the phenotype of infiltrating lymphocytes, we isolated IELs from the small intestine (SI) of *pIgR*^*−/−*^ and age-matched B6 mice. Similar to a previous report (Yamazaki et al. 2005), we found a significantly increased accumulation of SI-IELs in *pIgR*^*−/−*^ compared to B6 mice at 12 weeks of age, and the difference became especially prominent as mice aged to 34 weeks (Fig. 2a). Compared with B6 mice, SI-IELs in *pIgR*^*−/−*^ mice were enriched for αβ^+^ T cells (Fig. 2a). Notably, we observed that invariant αβ^+^ T cells, including MAIT cells (Fig. 2b) and NKT cells (Fig. 2c), were not enriched within the SI-IELs of *pIgR*^*−/−*^ mice, whereas conventional CD8α^+^αβ^+^ T cells (Fig. 2d) were the most abundant cell type infiltrating the small intestine.

Similar to their wild-type counterpart, CD8α^+^αβ^+^ T cells within the SI-IEL of *pIgR*^*−/−*^ mice co-expressed high levels of E-cadherin receptor CD103 and TCR engagement marker CD69 (Fig. 2e, top row), which are characteristic of tissue resident memory T cells (T_RM_). Enhanced accumulation of these cells likely resulted from ongoing stimulation from gut microbiota or metabolite in *pIgR*^*−/−*^ mice. Furthermore, we observed that *pIgR*^*−/−*^ mice carried an increased number of CD4^+^αβ^+^ T cells within SI-IELs (Fig. 2d). As mice became older, a sub-population of CD4^+^αβ^+^ T cells that expressed CD103 also upregulated CD8α expression (Fig. 2e, bottom row). We also tested for the presence of cytokines linked with inflammation in the supernatant from SI homogenate of 45-week old mice, as well as serum from 12-, 34- and 45-week old mice using cytometric bead array (CBA). IL-2, IL-4, IL-6, IL-10, IL-17A, IFN-γ and TNF were tested; only IFN-γ was detected in the SI of all *pIgR*^*−/−*^ mice (Fig. 2f). Flow cytometric analysis showed that an increased number of SI CD4^+^ αβ^+^ T cells produced IFN-γ after ex vivo re-stimulation with anti-CD3 antibody (data not shown), indicating that infiltrating αβ^+^ T cells most likely have a pathological effect in the gut of *pIgR*^−/−^ mice.

Collectively our results show a strong correlation between CD8α^+^αβ^+^ T cell infiltration and GIT pathology in *pIgR*^*−/−*^ mice, while a unique sub-population of CD4^+^CD8α^+^ double-positive (DP) αβ^+^ T cells may develop over time to neutralise the Th1-biased local environment in GIT.

### Delayed growth is associated with metabolic abnormalities in *pIgR*^−/−^ mice

**Fig. 3:**
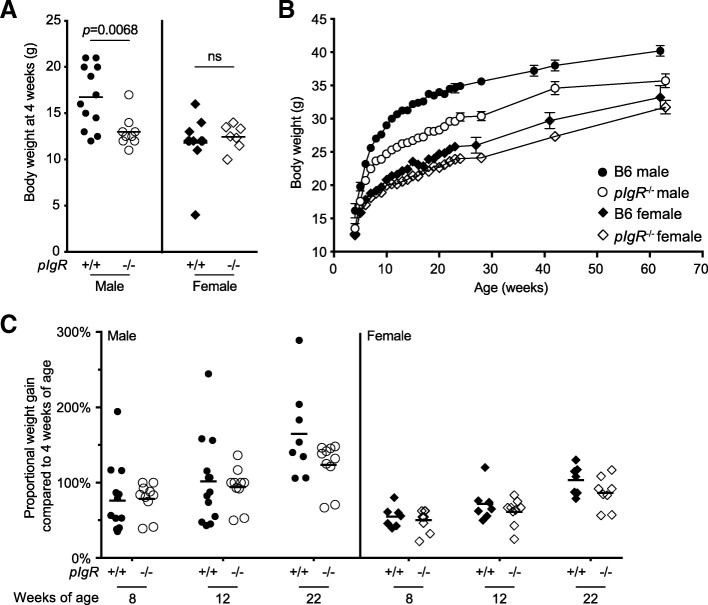
Effect of *pIgR* deletion on mouse body weight in B6 mice. Cohorts of age- and sex-matched male (closed circle, B6 *n* = 15; open circle, *pIgR*^−/−^ *n* = 10) and female (closed diamond, B6 n = 10; open diamond, *pIgR*^−/−^ n = 10) mice were weighed at indicated age. Shown are **a** body weight of individual mice at 4 weeks of age; and **b** mean ± SEM body weight over a 65-week time course. **c** Proportional weight gain is calculated as weight gained since 4 weeks of age as a percentage of 4-week body weight of the same mouse

We hypothesised that prolonged and escalating inflammation in the gut could impact on the integrity of the GI tract, which may in turn affect nutritional uptake and growth in *pIgR*^−/−^ mice. Intriguingly, we noticed that male *pIgR*^−/−^ mice appeared smaller and showed reduced body weight at the time of weaning (3–4 weeks of age) compared to their wild-type counterpart (Fig. 3a), an apparent delay in growth that may at least in part be attributable to reduced IgA level in the maternal breast milk (Rogier et al. 2014). To determine whether the lack of SAbs has lasting effect on growth into the adulthood, we established a large cohort of male and female B6 and *pIgR*^−/−^ mice and monitored their body weight from 4 weeks of age. We observed that the *pIgR*^−/−^ mice maintained a smaller body weight compared to age- and sex-matched wild-type controls at each time point taken, a trend that was most pronounced in the male animals (Fig. 3b), although the percentage weight gain since weaning at 4 weeks of age remained comparable between the genotypes for both sexes (Fig. 3c). This indicates that the deficiency of SAbs leads to a sustained but not exacerbated delay in weight building over an extended period of time.

**Fig. 4:**
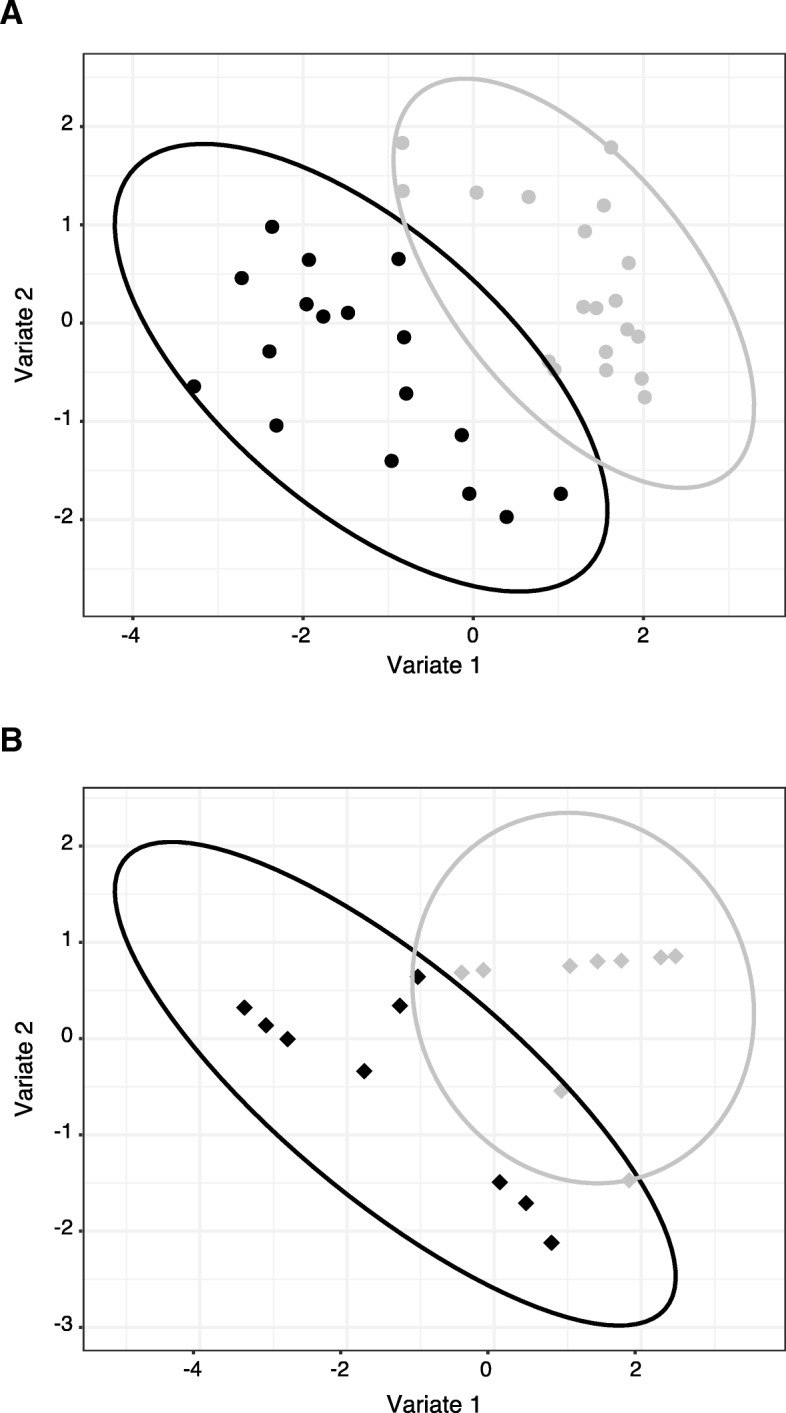
Scores plot analysis of metabolite profiles in urine of B6 and *pIgR*^*−/−*^ mice. GC-MS was performed on urine samples to determine the profile of metabolites excreted in the urine of **a** male and **b** female B6 and *pIgR*^−/−^ mice. To control for sample variation due to urine volume and concentration, the annotated urine metabolites were normalized by Mean Absolute Deviation (MAD). Data was analyzed by sPLS-DA and scores plot, with 95% confidence intervals of the sample space highlighted by elliptical boundaries

We next examined whether nutritional intake was altered in *pIgR*^−/−^ mice that showed reduced growth. GC-MS was used to determine the urinary concentrations of 42 specific metabolites, and sparse partial least square discriminant analysis (sPLS-DA) was performed to demonstrate whether urine samples, based on the profile of these 42 metabolites, segregated according to the *pIgR* genotype. As shown in the scores plots, which represent the distribution of samples in multivariate space, a clear separation between groups of male *pIgR*^−/−^ and B6 mice was observed (Fig. 4a), and a similar trend was also seen with urine samples from female mice (Fig. 4b). These results indicate that deficiencies in SAbs is correlated with metabolic abnormalities in *pIgR*^−/−^ mice.

**Fig. 5:**
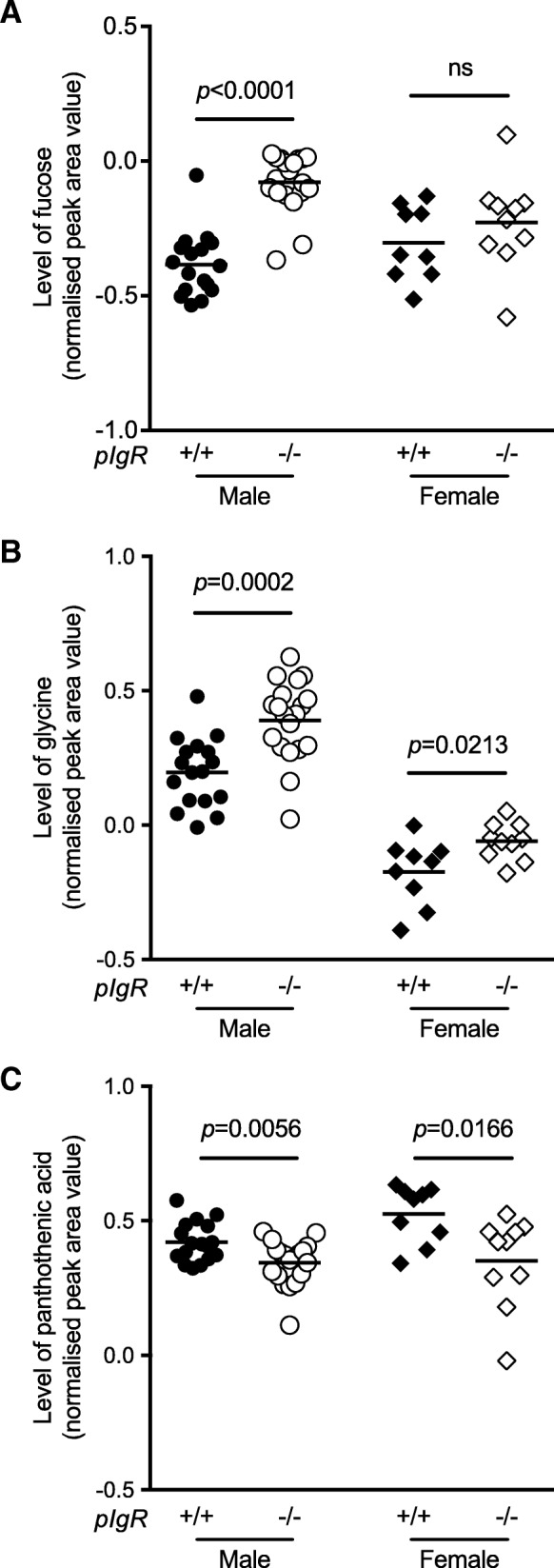
Specific metabolites in the urine of B6 and *pIgR*^−/−^ mice. GC-MS was performed on samples to determine the profile of metabolites excreted in urine of male (closed circle, B6 *n* = 17; open circle, *pIgR*^−/−^ *n* = 19) and female (closed diamond, B6 *n* = 9; open diamond, *pIgR*^−/−^ n = 10) mice. Samples were urease treated, ethanol extracted and analysed by GC-MS with *myo*-inositol as internal control and individual sugars quantitated using a standard sugar mix to determine response factors. Normalized peak area values are shown for **a** fucose, **b** glycine and **c** pantothenic acid. Unpaired *t*-test was used for statistical analyses and *p*-values are shown, ns indicates *p-*value > 0.05

To identify metabolites responsible for the group separation between B6 and *pIgR*^−/−^ mice, we used Student’s *t*-test to compare the abundance of individual urinary metabolites between B6 and *pIgR*^−/−^ mice. Using this approach, we identified 20 urinary metabolites that were differentially presented between *pIgR*^−/−^ mice and their wild-type counterparts in at least one sex, as highlighted in Table 1. This includes several compounds that are directly or indirectly involved in the tricarboxylic acid (TCA) cycle, such as citric acid, cis-aconitate, succinic acid and Vitamin B5 (pantothenic acid). In particular, pantothenic acid is required for the synthesis of coenzyme A and hence acetyl CoA; consistent with a reduction in pantothenic acid, both acetyl CoA and propanoate were decreased in the urine of *pIgR*^−/−^ mice. Three urinary metabolites, fucose, glycine and pantothenic acid, showed the strongest discrepancy between *pIgR*^−/−^ and B6 mice (Fig. 5). The level of fucose and glycine was elevated, whereas pantothenic acid was reduced in the mice lacking secretory antibodies and, consistent with other indicators of pathology, the difference was most significant in male mice.

**Table 1:**
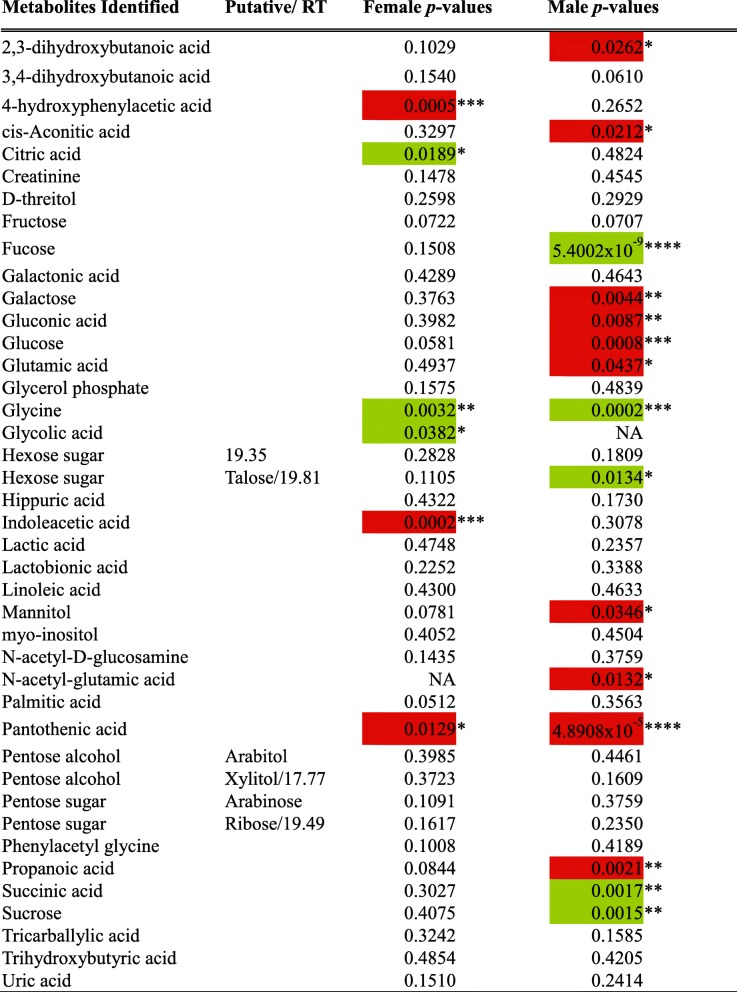
Compounds identified in urine metabolic profiles of B6 and *pIgR*^−/−^ male & female mice.

Statistical analysis of data normalised by MAD was performed using Student’s *t*-test, and *p*-values are given. Statistically significant differences where *pIgR*^−/−^ metabolite values were greater than B6 are highlighted in green, and decreases are highlighted in red. Statistically significant *p*-values are highlighted by *p < 0.05, **p < 0.01, ***p < 0.001 and ****p < 0.0001

### Increased epithelial permeability in *pIgR*^−/−^ mice as shown by altered ratios of sugar probes excreted in the urine

The detection of potential microbiota-derived metabolites in the urine of *pIgR*^−/−^ mice suggested increased epithelia permeability through the loss of SAbs in *pIgR*^−/−^ mice. To measure GIT permeability, we adapted the method described by Meddings & Gibbons (Meddings and Gibbons 1998) that utilised four sugar probes: sucrose, lactulose, mannitol and sucralose, which are absorbed in different regions of the GIT. The sugars were fed to mice and permeability was assessed by measuring excretion ratios (i.e. ingested versus excreted) in the urine. The presence of urinary sucrose is used as an indicator of increased stomach and duodenal permeability, lactulose and mannitol can indicate increased permeability of the small intestine, and sucralose can be used to assess all regions of the gut, including the large bowel.

**Fig. 6:**
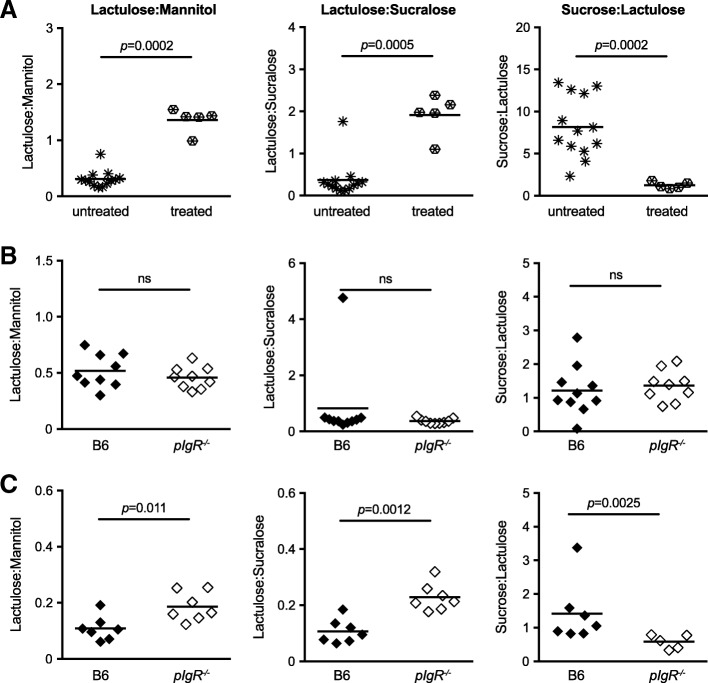
Gastrointestinal permeability of older adult mice to sugar probes. Gastrointestinal permeability was measured as altered ratios of sugar probes excreted in the urine. **a** B6 mice were treated with 5 mg aspirin two hours prior to feeding with sugar probes (open star sign, untreated *n* = 14; closed star sign, treated *n* = 6). **b** 10-week old or **c** 22-week old female B6 (closed diamond) or *pIgR*^−/−^ (open diamond) mice were given an oral dose of sugar probes containing sucrose, lactulose, sucralose and mannitol. The concentration of sugar probes in urine was collected 24-h post feeding and analysed by GC-MS. Data are represented as the ratio of sugar probe concentrations within samples and the horizontal bar represents the mean of each group. Statistical analysis was performed using the Mann Whitney *U-*test, ns indicates *p* > 0.05. Data are pooled from two independently performed experiments

To confirm that this approach can detect GI damage, we first fed wild-type B6 mice with a dose of 5 mg aspirin to induce GIT permeability, two hours prior to oral gavage with the four sugars. Twenty-four hours following treatment, mice that received aspirin showed pathology in the GIT (data not shown). GC-MS analysis of urine samples revealed a distinct change in the ratio of sugar probes after aspirin treatment (Fig. 6a), such that increased permeability in the small intestine was associated with increased ratios of lactulose to mannitol and lactulose to sucralose, whereas increased permeability in the large intestine led to decreased ratio of sucrose to lactulose similar to described before (Meddings and Gibbons 1998).

To determine the effect of SAbs on GIT permeability, age-matched *pIgR*^−/−^ and B6 wild-type mice were fed a bolus dose of sugar probes by oral gavage and urine samples were collected 24 h post-feeding. At 10-weeks of age, there was no difference in the sugar ratios between age-matched B6 and *pIgR*^−/−^ mice (Fig. 6b), suggesting that the GIT barrier is not compromised in young *pIgR*^−/−^ mice. In older *pIgR*^−/−^ mice (22 weeks of age), there was a significant increase in the ratio of lactulose to mannitol and lactulose to sucralose compared to B6 mice (Fig. 6c), indicating that *pIgR*^−/−^ mice show increased epithelial permeability in the small intestine. Similarly, we observed a decrease in the ratio of sucrose to lactulose and sucralose to mannitol in *pIgR*^−/−^ mice compared with B6 mice (Fig. 6c), indicating increased epithelial permeability in the large intestine of *pIgR*^−/−^ mice. There was no change in the ratio of sucrose to mannitol between B6 and *pIgR*^−/−^ mice that indicate stomach permeability (data not shown). Collectively, these results suggest that ageing *pIgR*^−/−^ mice have demonstrably greater permeability in the small and large intestines where cumulative pathology (Figs. 1 and 2) has been observed.

## Discussion

The mucosal immune system of the GIT plays a key role in preventing infection and damage by pathogenic organisms and their toxins. In this environment, the damage caused by immune responses to colonising microbiota and environmental molecules must be minimised, whilst also allowing the primary function of nutrient absorption to occur. The pIgR is expressed on the basolateral surface of columnar epithelial cells and is an essential component of the antibody secretion pathway. Studies utilising *pIgR*^*−/−*^ mice have been used to support the hypothesis that pIgR and SAbs are important for maintaining immune homeostasis of the mucosal lumen, and limiting mucosal and systemic immune responses to non-pathogenic environmental antigens (such as normal flora and food), through antigen ‘excretion’, a hypothesis first developed by Michael Lamm et al. in 1991 (Kaetzel et al. 1991). Under the excretion hypothesis, failure to excrete antigen can result in enhanced immune activation. We have shown that transgenic CD4^+^ T cells specific for ovalbumin proliferated to a higher extent in *pIgR*^−/−^ mice compared with B6 mice after oral feeding of ovalbumin (Sait et al. 2007), suggesting there may be impaired tolerogenic mechanisms in the absence of SAbs. It has also been reported that, in the absence of pIgR and SAbs, B6 mice have increased uptake of gut commensal bacteria into the mLNs, and this is associated with higher levels of antibodies specific to commensal bacteria (Sait et al. 2007; Johansen et al. 1999).

Previous studies have highlighted the role of SAbs in maintaining the integrity of the GIT, typically analysed using young mice, little was known about the long-term effect that low-grade but persistently increased gut permeability may have on the general health of the individual. In this study, we followed the development of B6 and isogenic mice deficient for SAbs (*pIgR*^*−/−*^) for more than 12 months. The delayed gain in body weight seen in *pIgR*^*−/−*^ mice was most obvious in males and was observed from 4 weeks of age, although signs of gross pathology, such as increased lymphocytic infiltration, damage to the epithelial layer of the GIT and increased permeabilities to sugar probes, became increasingly more apparently with age. This indicates a slow but cumulative effect that the absence of SAbs has on the health of GIT under homeostasis. Reduced weight gain can be due to reduced nutrient intake or increased metabolism, or both, and is often associated with an increase in GI permeability and/or inflammation of the gut, such as occurs in HIV enteropathy during AIDS (Bjarnason et al. 1996; Murphy et al. 1999). We show that this delay in growth was associated with an altered urine metabolome; in particular, a group of 20 metabolites differentiated the two genotypes of animals in a multivariate analysis. This effect was again the strongest in male mice, which also had a stronger phenotype for delayed growth. A number of the metabolites with altered concentrations in *pIgR*^*−/−*^ mice were components of the TCA cycle, e.g. a deficiency in pantothenic acid (Vitamin B5), required for the synthesis of coenzyme A. Since many of these metabolites can be derived from both eukaryotic and prokaryotic metabolism, it is not possible to identify their source.

To investigate whether these changes in the urine metabolome were associated with functional changes in GIT function, we measured GIT permeability using a modified sugar feeding assay. This assay was validated following aspirin-induced GIT damage, which has previously been shown to alter GI permeability to sugar probes in rodents (Meddings and Gibbons 1998; Worthington et al. 1978; Alnadjim et al. 2002). Using this assay, we demonstrated that aged mice lacking SAbs, but not younger mice, had demonstrably increased permeability, a phenomenon that was not observed in wild-type animals. The increased permeability was associated with an increase in immunopathology. The effect was most marked in the duodenum and colon and characterized by a reduction in mucin, larger nuclei in crypt epithelium and increased lymphocyte numbers.

The increased gut permeability seen in *pIgR*^−/−^ animals is also observed in other diseases and conditions affecting the GIT, which present as significantly delayed growth or a failure to thrive (Arrieta et al. 2006). These include gastrointestinal ‘allergy’ (Zanjanian 1976), Celiac disease (Vogelsang et al. 1998), Crohn’s disease and ulcerative colitis (Munkholm et al. 1994; Söderholm et al. 1999a; Söderholm et al. 1999b; Hollander 2002), irritable bowel syndrome (Barau and Dupont 1990), type 1 diabetes (Secondulfo et al. 2004) and infection (Ding et al. 2004). These studies suggest that immunodeficiencies that are characterized by impaired B cell function and reduced levels of IgA and IgM (e.g. common variable immune deficiency, CVID), and which are associated with mucosal inflammatory and autoimmune diseases (Cunningham-Rundles 2010) that might result from the loss of the antigen ‘excretion’ pathway (Strugnell and Wijburg 2010), a pathway facilitated by SAbs.

We noted that lymphocytic infiltration in *pIgR*^−/−^ mice was enriched for CD8α^+^αβ^+^ T cells of an apparent resident memory phenotype (CD103^+^CD69^+^), and the enrichment was enhanced with age. We speculate that excess accumulation of these cells in the GIT of *pIgR*^−/−^ mice most likely occurred in response to ongoing stimulation from gut microbiota and metabolites. In addition, these CD8α^+^αβ^+^ T_RM_ may be the primary mediator of GIT pathology due to their capacity of producing cytotoxic granzyme B (Casey et al. 2012). We also observed the infiltration of CD4/CD8 double positive (DP) αβ^+^ T cells. Peripheral CD4/CD8 DP T cells have been detected in a number of disease models and across several species (reviewed in (Overgaard et al. 2015)). The role of these cells appears to be disease-specific, ranging from pro-inflammatory in cancer models, to suppressive Treg functions in the gut (Das et al. 2003). Although the majority of 34-week old *pIgR*^*−/−*^ mice harboured an increased number of CD4/CD8 DP T cells, the absolute number of these cells varies widely between individual animals. Whether the expansion of these cells dampens, or further contributes to immune pathology, remains unclear.

## Conclusions

Our observations suggest that the absence of SAbs in otherwise healthy animals is associated with increased inflammation of the epithelium, increased GIT permeability and altered metabolism as the consequence, leading to delayed growth. The lymphocytes that accumulate in the epithelia appear phenotypically to be conventional CD8^+^ T cells, not NKT cells or MAIT cells. MAIT cells are activated by riboflavin metabolites produced by the microbiota (Corbett et al. 2014). These studies, conducted under SPF environment, suggest that the pIgR and SAbs play an important role in maintaining homeostasis of the uninfected GIT and limit mucosal and systemic immune responses to notionally non-pathogenic environmental antigens, perhaps in concert with PAMPs, such as LPS, CpG DNA, peptidoglycan (Mills 2011) released from the microbiota (Wang et al. 2015). Urine metabolite analysis revealed several potential biomarkers for failure to thrive, including increased succinate levels. Notably, severely malnourished children also demonstrate elevated succinate levels in their urine (Capo-Chichi et al. 2000). These observations suggest that cases of human ‘failure to thrive’ might result from micro-inflammation of the epithelia resulting from loss of antibody secretion, warranting further investigation.

## Additional file

Additional file 1: **Figure S1** IgA and IgG levels in the serum and faeces indicate selective depletion of SIgA in *pIgR*^*−/−*^ mice. (DOCX 576 kb)

## Abbreviations

Ab: Antibody
ASC: Antibody-secreting cells
B6: C57BL/6
CBA: Cytometric bead array
CD: Cluster of differentiation
CVID: Common variable immunodeficiency
DL: Detection limit
DNA: Deoxyribonucleic acid
DP: Double-positive
FACS: Fluorescence-activated cell sorting
GC-MS: Gas chromatography-mass spectrometry
GIT: Gastrointestinal tract
H&E: Hematoxylin and eosin
IEL: Intraepithelial lymphocytes
Ig: Immunoglobulin
LP: Lamina propria
LPS: Lipopolysaccharide
MAD: Mean absolute deviation
MAIT: Musoca-associated invariant T
mLNs: mesenteric lymph nodes
NKT: Natural killer T
NOD: Nucleotide-binding oligomerisation domain
PAMP: Pathogen-associated molecular pattern
pIgR: Polymeric immunoglobulin receptor
SAb: Secretory antibody
SEM: Standard error of the mean
SI: Small intestine
SPF: Specific pathogen free
sPLS-DA: Sparse partial least square discriminant analysis
TCA: Tricarboxylic acid
Th: T helper
T_RM_: Tissue resident memory T
