# Supplementary material for: Mus musculus deficient for secretory antibodies show delayed growth with an altered urinary metabolome
Source: Mol Med. 2019 Apr 3;25:12. doi: 10.1186/s10020-019-0077-2 (PMC6446318; doi:10.1186/s10020-019-0077-2)
Supplement: Supplementary file 1 — Figure S1 IgA and IgG levels in the serum and faeces indicate selective depletion of SIgA in pIgR−/− mice. (DOCX 576 kb) [file 10020_2019_77_MOESM1_ESM.docx]

Additional file 1

**Figure S1. IgA and IgG levels in the serum and faeces indicate selective depletion of SIgA in *pIgR^-/-^* mice.** Faecal extracts and serum samples were obtained from naïve B6 (closed circle), *pIgR^-/-^* (open circle) and μMT (open square) mice, the concentration of total IgA (A, faecal extracts; B, serum samples) and IgG (C, faecal extracts; D, serum samples) was measured by ELISA. Symbols represent data from individual animals, and horizontal lines represent the geometric mean of each group. One-way ANOVA with Tukey post-tests was used to for statistical analysis, ns indicates *p-*value > 0.05. Data are representative of two to three independent experiments.
